# Upregulation of PGC-1α expression by Alzheimer’s disease-associated pathway: presenilin 1/amyloid precursor protein (APP)/intracellular domain of APP

**DOI:** 10.1111/acel.12183

**Published:** 2013-12-17

**Authors:** Ari Robinson, Sven Grösgen, Janine Mett, Valerie C Zimmer, Viola J Haupenthal, Benjamin Hundsdörfer, Christoph P Stahlmann, Yulia Slobodskoy, Ulrike C Müller, Tobias Hartmann, Reuven Stein, Marcus O W Grimm

**Affiliations:** 1 Department of Neurobiology, George S. Wise Faculty of Life Sciences, Tel Aviv UniversityRamat Aviv, Israel; 2 Neurodegeneration and Neurobiology, Saarland UniversityHomburg/Saar, Germany; 3 Department of Functional Genomics, Institute of Pharmacy and Molecular Biotechnology, Heidelberg UniversityHeidelberg, Germany; 4 Deutsches Institut für DemenzPrävention (DIDP), Saarland UniversityHomburg/Saar, Germany; 5 Experimental Neurology, Saarland UniversityHomburg/Saar, Germany; 6 Sagol School of Neuroscience, Tel Aviv UniversityTel Aviv, Israel; * Mina and Everard Goodman Faculty of Life Sciences, Bar-Ilan UniversityRamat-Gan, Israel

**Keywords:** Alzheimer’s disease, amyloid precursor protein processing, amyloid precursor protein intracellular domain, Fe65, PGC1-α, mitochondrial function

## Abstract

Cleavage of amyloid precursor protein (APP) by β- and γ-secretase generates amyloid-β (Aβ) and APP intracellular domain (AICD) peptides. Presenilin (PS) 1 or 2 is the catalytic component of the γ-secretase complex. Mitochondrial dysfunction is an established phenomenon in Alzheimer’s disease (AD), but the causes and role of PS1, APP, and APP’s cleavage products in this process are largely unknown. We studied the effect of these AD-associated molecules on mitochondrial features. Using cells deficient in PSs expression, expressing human wild-type PS1, or PS1 familial AD (FAD) mutants, we found that PS1 affects mitochondrial energy metabolism (ATP levels and oxygen consumption) and expression of mitochondrial proteins. These effects were associated with enhanced expression of the mitochondrial master transcriptional coactivator PGC-1α and its target genes. Importantly, PS1-FAD mutations decreased PS1’s ability to enhance PGC-1α mRNA levels. Analyzing the effect of APP and its γ-secretase-derived cleavage products Aβ and AICD on PGC-1α expression showed that APP and AICD increase PGC-1α expression. Accordingly, PGC-1α mRNA levels in cells deficient in APP/APLP2 or expressing APP lacking its last 15 amino acids were lower than in control cells, and treatment with AICD, but not with Aβ, enhanced PGC-1α mRNA levels in these and PSs-deficient cells. In addition, knockdown of the AICD-binding partner Fe65 reduced PGC-1α mRNA levels. Importantly, APP/AICD increases PGC-1α expression also in the mice brain. Our results therefore suggest that APP processing regulates mitochondrial function and that impairments in the newly discovered PS1/APP/AICD/PGC-1α pathway may lead to mitochondrial dysfunction and neurodegeneration.

## Introduction

Presenilin 1 (PS1) and presenilin 2 (PS2), derived from *PSEN1* and *PSEN2*, first identified as genes mutated in families with familial Alzheimer’s disease (FAD) (Cruts *et al*., 1996), form the catalytic component of the γ-secretase complex (Steiner, 2008; Li *et al*., 2009). This secretase, an intramembrane aspartyl protease, is critically involved in Alzheimer’s disease (AD) via proteolysis of the amyloid precursor protein (APP), which generates the pathogenic and amyloid plaque-forming amyloid-β (Aβ)_42_ peptide (Steiner, 2008; Hass *et al*., 2009). Importantly, cleavage of APP by the secretase complex also results in the generation of the APP intracellular C-terminal domain (AICD) (Weidemann *et al*., 2002). AICD has been proposed to act as a transcriptional regulator via a mechanism involving interaction with the adaptor protein Fe65 and the action of Tip60 (McLoughlin & Miller, 2008; Chang & Suh, 2010).

Mitochondria play a crucial role in many fundamental cellular processes, ranging from energy production and metabolism to apoptosis. Mitochondrial dysfunction is widely implicated in the pathogenesis of many neurodegenerative diseases, including AD (Ishii *et al*., 1997; Blass *et al*., 2000; Riemer & Kins, 2013). For example, AD pathology is associated with decreased expression and activity of proteins involved in mitochondrial bioenergetics, and mitochondrial dysfunction has also been observed in the triple transgenic mouse model for AD where it was shown to precede plaque formation (Yao *et al*., 2009). Mitochondrial dysfunction in AD may involve the action of APP and Aβ, as they were reported to target the mitochondria and cause mitochondrial dysfunction (Caspersen *et al*., 2005; Devi *et al*., 2006; Manczak *et al*., 2006).

Peroxisome proliferator-activated receptor-γ coactivator 1α (PGC-1α) is a transcriptional coactivator that regulates the transcription of nuclear respiratory factor (NRF) 1 and 2, leading to increased expression of mitochondrial transcription factor A and other nuclear-encoded mitochondrial proteins, for example ATP synthase subunits. Collectively, these PGC-1α effects culminate in the regulation of mitochondrial biogenesis and energy metabolism (Lin *et al*., 2005; Rodgers *et al*., 2008).

The mechanism(s) leading to mitochondrial dysfunction in AD is unknown. In view of the important role played by PSs and the amyloidogenic APP processing in AD, we hypothesize that the PSs/APP processing pathway regulates mitochondrial functionality. The present study examined the effect of this pathway on mitochondrion-associated features, for example energy metabolism and PGC-1α expression. Our results suggest that PS1 regulates the expression of PGC-1α by the γ-secretase-dependent APP cleavage product AICD. As a consequence, PSs deficiency leads to reductions in ATP level, oxygen consumption rate, and expression of PGC-1α target genes and proteins. Furthermore, PS1-FAD mutations reduce PS1’s ability to regulate PGC-1α mRNA levels.

## Results

### The effect of PS1 on mitochondrial-associated features

To examine the effect of PS1 on mitochondria, we compared mitochondrial features in PS1/PS2 double-knockout (DKO) mouse embryonic fibroblasts (MEFs) reconstituted with WT PS1 (PS1r) to those in DKO MEFs transfected with empty vector (herein PS1/2^−/−^). The advantage offered by this system is that the only apparent difference between these two cell lines is PS1 expression.

**Figure 1:**
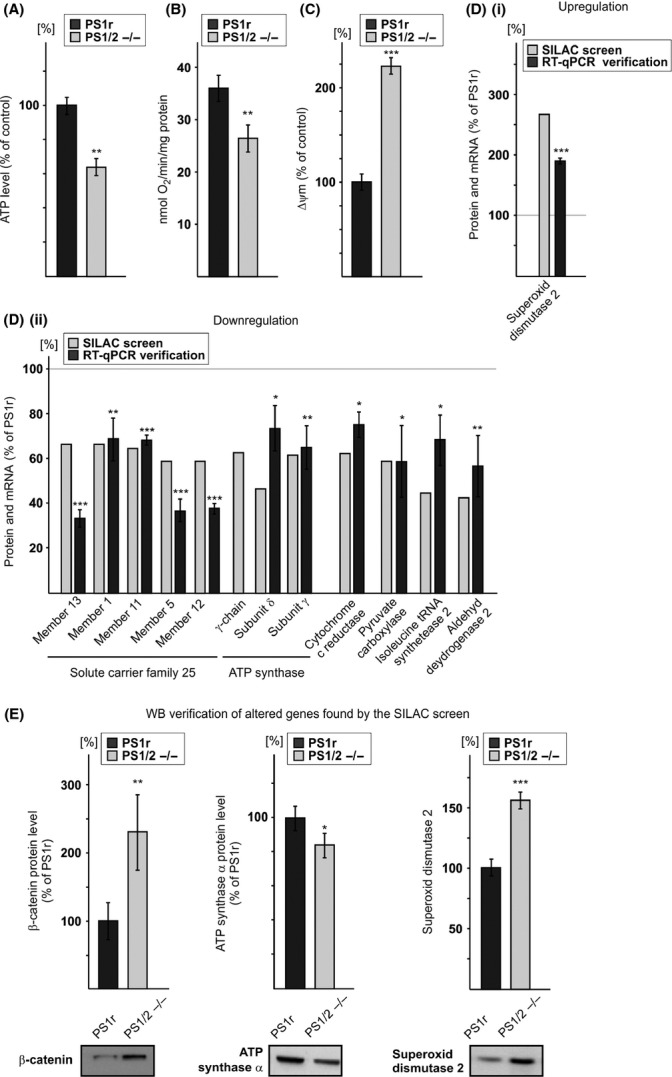
PS1 effects on the mitochondrial-related features. (A) ATP levels are lower in PS1/2^−/−^ than in PS1r mouse embryonic fibroblasts (MEFs). ATP levels were measured in triplicate using ATP bioluminescence assay kit. Values shown are means ± SEM (bars) (*n* = 5); **P < 0.01 (Student’s *t*-test). (B) Oxygen consumption rate is lower in PS1/2^−/−^ than in PS1r MEFs. Oxygen consumption rate of intact cells was measured as described in Experimental procedures. Data are expressed as nmol O_2_ min^−1^ mg^−1^ protein. Values shown are means ± SEM (bars) (*n* = 11); **P < 0.01 (Student’s *t*-test). (C) ΔΨm is higher in PS1/2^−/−^ than in PS1r MEFs. ΔΨm in intact cells was measured as described in Experimental procedures. Values shown are means ± SEM (bars) (*n* = 5); ***P < 0.001 (Student’s *t*-test). (D) upregulated (i) or downregulated (ii) expression of mitochondrial proteins and their corresponding mRNAs. PS1r and PS1/2^−/−^ MEFs were subjected to stable isotope labeling with amino acids in cell culture coupled to LC-mass spectrometry (MS)/MS analysis as described in Experimental procedures. The results are expressed as percentage of the average values of the light (PS1/2^−/−^) peptides from the average values of the heavy (PS1r) peptides of each protein. The proteins shown are mitochondrial proteins that were up- or downregulated in PS1/2^−/−^ MEFs compared to PS1r MEFs (1.5 cutoff). The expression of the mRNAs corresponding to the depicted proteins is shown adjacent to each of the corresponding protein. mRNA levels were measured by real-time reverse transcription quantitative polymerase chain reaction and normalized to β-actin. The results are expressed as percentage of PS1r. Values shown are means ± SEM (bars) (*n* > 5); *P < 0.05, **P < 0.01, and ***P < 0.001 (Student’s *t*-test). (E) β-catenin, ATP synthase subunit α, and superoxide dismutase 2 protein expression. Total cell extracts prepared from PS1/2^−/−^ and PS1r cells were subjected to SDS-PAGE followed by immunoblotting. The images shown are from representative experiments (*n* = 3–10). Immunoblots obtained from all independent experiments were scanned, and intensities of the different proteins assessed by densitometric analysis. Data are expressed as mean intensity values ± SEM (bars). *P < 0.05, **P < 0.01, ***P < 0.001 (Student’s *t*-test).

In the first set of experiments, we examined the effect of PS1 on three mitochondrial-related energy metabolism features: ATP, oxygen consumption, and mitochondrial membrane potential (ΔΨm). The results show that total ATP levels (Fig. 1A) and oxygen consumption rate (Fig. 1B) were significantly lower, and ΔΨm [measured by tetramethylrhodamine methyl ester (TMRM) probe] was significantly higher (Fig. 1C) in PS1/2^−/−^ MEFs compared to PS1r MEFs. Collectively, these results suggest that PS1 is involved in mitochondrial energy metabolism.

Next, the proteome of mitochondrial-enriched subcellular fractions of PS1r and PS1/2^−/−^ cells was determined using stable isotope labeling with amino acids in cell culture (SILAC)/mass spectrometry (MS)/MS. This approach identified 545 proteins, of which 25% were mitochondrial proteins. Examination of the proteins exhibiting differential expression in the PS1r and PS1/2^−/−^ samples using a 1.5-fold cutoff revealed down- and upregulation of 52 and 72 proteins, respectively, in the PS1/2^−/−^ sample compared to the PS1r sample. Among these, one mitochondrial protein was upregulated, and 12 were downregulated (Fig. 1D). The SILAC/MS/MS results also revealed 2.94-fold higher β-catenin expression in PS1/2^−/−^ cells than in PS1r cells. β-Catenin has been shown to accumulate in PS1-deficient cells such as PS1/PS2-DKO MEFs (Boo *et al*., 2009) because of the absence of its PS1-mediated degradation (Murayama *et al*., 1998; Kang *et al*., 1999). Analysis of β-catenin expression by immunoblotting (Fig. 1E) validated the SILAC/MS/MS results and showed 2.3-fold higher β-catenin expression in PS1/2^−/−^ MEFs than in PS1r MEFs. The detection of β-catenin as a PS1-regulated protein in the PS1r/PS1/2^−/−^ cell system and the SILAC methodology serves as a proof of concept for this system’s ability to identify PS1-regulated proteins. Next, the expression levels of the mRNAs corresponding to the PS1-regulated mitochondrial proteins identified by SILAC/MS/MS were determined by real-time reverse transcription quantitative polymerase chain reaction (RT-qPCR) analysis. As shown in Fig Di and Dii, the PS1-regulated profile observed at the protein level (SILAC/MS/MS) was recapitulated at the mRNA level, confirming the PS1-regulated manner of the proteins identified by SILAC/MS/MS. To further confirm this notion, we examined the expression level of superoxide dismutase 2 and ATP synthase subunit α proteins identified by SILAC/MS/MS to be up- or downregulated, respectively, in PS1/2^−/−^ MEFs. Figure 1(E) shows that in line with the SILAC/MS/MS results, superoxide dismutase 2 protein level was significantly higher and ATP synthase subunit α lower in PS1/2^−/−^ MEFs compared to PS1 MEFs.

Among the mitochondrial proteins whose expression was discovered by SILAC/MS/MS analysis to be upregulated (cutoff 1.5) were ATP synthase subunit γ precursor (γ chain), ATP synthase subunit γ, and ATP synthase subunit δ. As F_1_F_O_ ATP synthase, the main source of cellular ATP, is a multisubunit protein, we examined whether PS1 also regulates other ATP synthase subunits. The SILAC/MS/MS analysis detected additional four ATP synthase subunits (O, b isoform 1, β, and α) (Table S1, Supporting Information). Interestingly, the level of these ATP synthase subunits was also lower (although to a lesser extent) in PS1/2^−/−^ than in PS1r cells (75–87% of PS1r).

**Figure 2:**
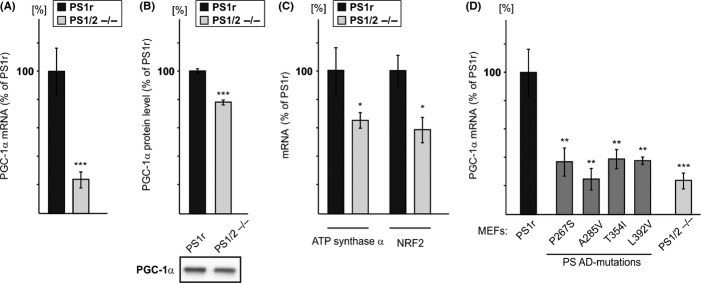
The effect of PS1 on the expression of PGC-1α and PGC-1α target genes. (A) PGC-1α mRNA level is lower in PS1/2^−/−^ than in PS1r clones. Total RNA was prepared from four independent PS1r clones and three independent PS1/2^−/−^ clones, each sample in triplicate. PGC-1α mRNA levels were measured by real-time reverse transcription quantitative polymerase chain reaction (RT-qPCR) and normalized to β-2-microglobulin (B2M). The value of each measurement was normalized to one of the samples of the PS1/2^−/−^ clones, and the results are expressed as fold increase relative to this clone. Values shown are means ± SEM (bars) (*n* = 9 and 12 for PS1/2^−/−^ and PS1r clones, respectively); ***P < 0.001 relative to PS1/2^−/−^ cells (Student’s *t*-test). (B) PGC-1α protein expression. Total cell extracts were prepared from PS1/2^−/−^ and PS1r cells, and the expression of PGC-1α protein (lower panel) was determined. Shown immunoblotting results are from a representative experiment. Quantification of immunoblots obtained from all independent experiments is shown in the upper panel. Data are expressed as mean values ± SEM (bars) (*n* = 5). ***P < 0.001 (Student’s *t*-test). (C) ATP synthase subunit α and NRF2 mRNA levels are lower in PS1/2^−/−^ than in PS1r clones. Total RNA was prepared from three independent PS1r clones and two independent PS1/2^−/−^ clones, each clone in triplicate. ATP synthase subunit α and NRF2 mRNA levels were measured by RT-qPCR and normalized to B2M. Values shown are means ± SEM (bars) (*n* = 6 and 9 and *n* = 6 and 9 for ATP synthase subunit α and NRF2 in PS1/2^−/−^ and PS1r, respectively); *P < 0.05 relative to PS1/2^−/−^ cells (Student’s *t*-test). (D) Effect of PS1-familial Alzheimer’s disease mutations P267S, A285V, T354I, and L392V on PGC-1α mRNA expression. Total RNA was prepared from the indicated mouse embryonic fibroblasts (MEF) clones and subjected to RT-qPCR analysis. The analysis consists of one clone for each of the P267S, A285V, and L392V MEFs and two independent clones for the T354I MEFs, each clone in triplicate. For comparison, the histogram also includes the data presented in (A) (PS1r and PS1/2^−/−^). Presented data were all normalized to the same PS1/2^−/−^ clone as in (A). Values shown are means ± SEM (bars) [*n* = 3 for all clones except for clone T354I (*n* = 6)]; **P < 0.01, ***P < 0.001 relative to PS1r (Student’s *t*-test). No significant difference was observed between P267S, A285V, T354I, or P436Q and PS1/2^−/−^ (Student’s *t*-test).

The observed downregulation of expression of all six ATP synthase subunits detected by the SILAC/MS/MS approach in the PS1/2^−/−^ sample suggests that a common mechanism regulates the expression of these proteins. The expression of respiratory chain proteins, including the different subunits of ATP synthase, is regulated by the transcription factors NRF1 and 2 (Kelly & Scarpulla, 2004), which act together with PGC-1α coactivator (Scarpulla, 2008). We therefore speculated that the coordinated PS1-dependent regulation of the different subunits of ATP synthase is mediated by PGC-1α. Examination of PGC-1α mRNA levels in several PS1r and PS1/2^−/−^ clones by RT-qPCR analysis revealed its substantially and significantly lower expression in PS1/2^−/−^ vs. PS1r clones (Fig. 2A). Next, we examined PGC-1α protein levels in PS1r and PS1/2^−/−^ MEFs by immunoblotting. Analysis of the relative intensity of PGC-1α protein (~90 kDa) in PS1r and PS1/2^−/−^ MEFs revealed a decrease in expression in PS1/2^−/−^ MEFs compared to PS1r MEFs (20%) (Fig. 2B). To examine whether PS1 affects also the expression of PGC-1α target genes, we examined mRNA levels of ATP synthase subunit α and NRF2, two known targets of PGC-1α, by RT-qPCR analysis. As shown in Fig. 2(C), the ATP synthase α and NRF2 mRNA levels were significantly lower in the PS1/2^−/−^ clones compared to the PS1r clones. Collectively these results suggest that PS1 regulates expression of PGC-1α and its target genes.

To gain insight into the mechanism governing PS1 regulation of PGC-1α and to relate this effect to AD, we next examined the effect of PS1-FAD mutations on PGC-1α expression. To this end, PGC-1α mRNA levels were assessed in PS1/2^−/−^ MEFs reconstituted with each of the following human PS1-FAD-associated mutations: P267S, A285V, T354I, or L392V. As shown in Fig. 2(D), PGC-1α mRNA levels in P267S, A285V, T354I, and L392V MEFs were found to be lower than in PS1r MEFs, suggesting that these PS1-FAD mutations impair the mutant proteins’ ability to regulate PGC-1α mRNA expression.

### The effects of APP, Aβ, and AICD on PGC-1α expression

**Figure 3:**
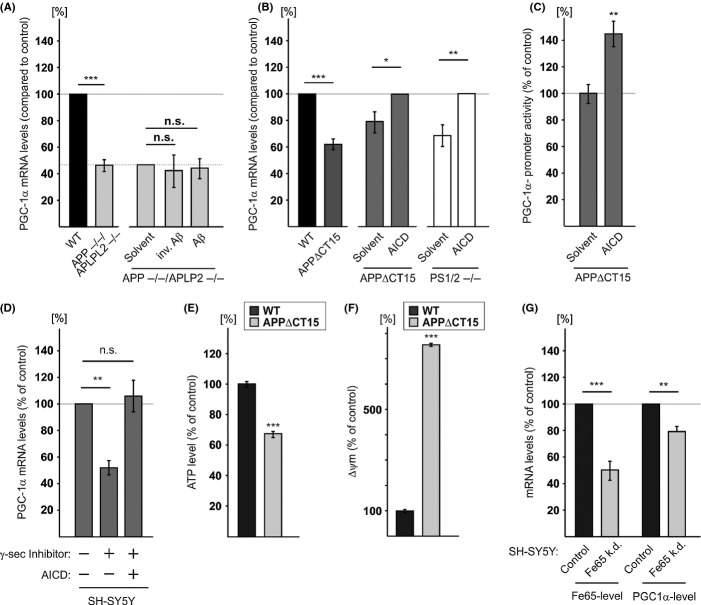
PGC-1α expression is regulated by amyloid precursor protein (APP) and its γ-secretase cleavage product APP intracellular domain (AICD). (A) APP/APLP2 deficiency reduces PGC-1α mRNA levels by a mechanism that cannot be rescued by exogenous Aβ. Total RNA was prepared from WT and *APP/APLP2*^−/−^ mouse embryonic fibroblasts (MEFs), subjected to real-time reverse transcription quantitative polymerase chain reaction (RT-qPCR) analysis, and normalized to β-actin. Each clone was analyzed in triplicate. Values shown are means ± SEM (bars); ***P < 0.001 relative to WT MEF (Student’s *t*-test). *APP/APLP2*^−/−^ MEFs were incubated with H_2_O (solvent), Aβ or inverted Aβ peptides for 9 days and then PGC-1α mRNA levels were determined. The values presented for *APP/APLP2*^−/−^ MEFs treated with solvent control were normalized to the values of untreated *APP/APLP2*^−/−^ MEFs as indicated by the dashed line. Data are expressed as means ± SEM (bars) (*n* = 5); n.s. = not significant (Student’s *t*-test). (B) Effect of AICD on PGC-1α mRNA levels. APP∆CT15 MEFs or double-knockout MEFs (PS1/2^−/−^) were incubated with AICD peptide or with H_2_O (solvent) for 9 days, and then PGC-1α mRNA levels were determined. Values shown are means ± SEM (bars) The values presented for the AICD-treated cells (APP∆CT15 and PS1/2^−/−^ MEFs) were normalized to WT MEFs as indicated by the dashed line. (*n* = 5); *P < 0.05, **P < 0.01, and ***P < 0.001 (Student’s *t*-test). (C) AICD increases PGC-1α promoter activity in APPΔCT15 MEFs. APPΔCT15 MEFs were transfected with the different luciferase reporter plasmids and then incubated with AICD or H_2_O (solvent) as described in Experimental procedures. Values shown are means ± SEM (bars) (*n* = 4); **P < 0.01 (Student’s *t*-test). (D) Exogenously supplemented AICD bypassed the inhibitory effect of γ-secretase inhibition. SH-SY5Y cells were incubated with the γ-secretase (γ-sec) inhibitor L-658.458 or solvent (DMSO) in the presence or absence of AICD as described in Experimental procedures. PGC-1α mRNA levels were analyzed by RT-qPCR. Values shown are means ± SEM (bars) (*n* = 12); **P < 0.01 and n.s. = not significant, (Student’s *t*-test). (E) ATP level is lower ***P < 0.001 and mitochondrial membrane potential (Δψm) (F) is higher in APPΔCT15 MEFs compared to WT MEFs. ATP level and Δψm were determined as described in Fig. 1(A,C). Values shown are means ± SEM (bars) (*n* = 5); ***P < 0.001 (Student’s *t*-test). (G) The effect of Fe65 knockdown on PGC-1α mRNA levels. Total RNA was prepared from SH-SY5Y-Fe65KD (Fe65 k.d.) and SH-SY5YCon (control) cells and analyzed for Fe65 and PGC-1α mRNA levels by RT-qPCR analysis. Values shown are means ± SEM (bars); (*n* = 5); **P < 0.01, ***P < 0.001 (Student’s *t*-test).

Familial Alzheimer’s disease-associated PS1 mutations are thought to promote AD by modulating γ-secretase activity, particularly on APP, producing a higher ratio of Aβ42/40 and a lower AICD level (Walker *et al*., 2005; Wiley *et al*., 2005; Bentahir *et al*., 2006; Kumar-Singh *et al*., 2006). Moreover, the PS1-FAD mutations tested here and shown to attenuate the effect of PS1 on PGC-1α are known to decrease γ-secretase activity (Duering *et al*., 2005; Walker *et al*., 2005; Wiley *et al*., 2005; Bentahir *et al*., 2006; Kumar-Singh *et al*., 2006). Therefore, we speculated that the PS1 effect on PGC-1α expression is mediated via APP and its γ-secretase cleavage products, Aβ and AICD. To assess the effect of APP, we employed MEFs deficient in APP and APLP2 (*APP/APLP2*^−/−^). PGC-1α mRNA levels in *APP/APLP2*^−/−^ MEFs were significantly lower (53% reduction) than in WT MEFs (Fig. 3A), suggesting that APP or its cleavage products are involved in regulation of PGC-1α expression. To test whether APP regulates PGC-1α expression via Aβ, we examined whether supplementation of Aβ peptides to *APP/APLP2*^−/−^ MEFs would enhance PGC-1α expression. *APP/APLP2*^−/−^ MEFs were incubated with a mixture of Aβ_42_ (1 ng mL^−1^) and Aβ_40_ (10 ng mL^−1^) or a mixture of inverted Aβ (42 aa, 1 ng mL^−1^; 40 aa 10 ng mL^−1^) peptides: neither Aβ nor the inverted Aβ peptides had any significant effect on PGC-1α expression (Fig. 3A), although the Aβ peptides were taken up by the *APP/APLP2*^−/−^ MEFs (Fig. S1, Supporting Information). These results suggest that Aβ peptides do not affect PGC-1α expression. To test whether AICD, the other APP cleavage product, is involved in PGC-1α expression regulation, we employed APPΔCT15 MEFs. These cells were derived from mice in which the endogenous APP gene had been replaced with an APP variant lacking the last 15 aa of APP harboring the YENPTY motif (Ring *et al*., 2007), a motif known to mediate AICD activity (Schettini *et al*., 2010). PGC-1α mRNA levels were significantly lower (40%) in APPΔCT15 than in WT MEFs (Fig. 3B), suggesting that the absence of AICD impaired APP’s ability to regulate PGC-1α. Therefore, we examined whether addition of AICD peptide to APPΔCT15 MEFs or PS1/2^−/−^ MEFs would enhance PGC-1α expression. Incubation of APPΔCT15 MEFs or PS1/2^−/−^ MEFs with 2 μm AICD peptide induced a significant increase (25% or 47% respectively) in PGC-1α mRNA levels compared to APPΔCT15 MEFs or PS1/2^−/−^ MEFs incubated with solvent alone (Fig. 3B). Immunofluorescence analysis revealed that the AICD peptide was taken up by the cells (Fig. S2, Supporting Information).

To further assess the impact of AICD on PGC-1α expression and to determine whether such an effect is mediated via PGC-1α promoter activity, the effect of AICD on the activity of PGC-1α promoter was examined in APPΔCT15 MEFs. APPΔCT15 MEFs were transfected with PGC-1α-Luc (a promoter–reporter construct of PGC-1α) (Handschin *et al*., 2003) or promoter-less pGL3-basic construct (to determine basal activity) and incubated with AICD or solvent control. For assessing transfection efficiency, the cells were co-transfected with pRL-CMV (for constitutive expression of Renilla luciferase). Analysis of the relative luciferase activity calculated by normalization of firefly to Renilla luciferase activity revealed that the activity of the PGC-1α promoter in AICD-treated APPΔCT15 MEFs was significantly higher compared to the solvent-treated cells (Fig. 3C).

To further support the notion that the γ-secretase cleavage product AICD regulates PGC-1α expression, we examined whether inhibition of γ-secretase activity would inhibit PGC-1α expression. SH-SY5Y cells were incubated with the γ-secretase inhibitor L-658.458. In line with the effect of PSs deficiency, treatment with L-658.458 significantly reduced PGC-1α mRNA levels (Fig. 3D). Importantly, addition of AICD peptide to γ-secretase inhibitor-treated SH-SY5Y cells bypassed the effect of the inhibitor and completely restored PGC-1α mRNA levels (Fig. 3D). Notably, in contrast to the effect of exogenously added AICD peptide on AICD-lacking cells (Fig. 3B), such treatment had only a slight and nonsignificant effect on PGC-1α expression (Fig. S3, Supporting Information) in SH-SY5Y cells. This result suggests that the concentration of endogenous AICD in SH-SY5Y cells was sufficient to promote maximal activity, and thus, the exogenously added AICD had no or only a minor effect.

Next, we examined whether in analogy to PSs deficiency, lack of AICD would affect mitochondrial energy metabolism. To this end, ATP level and ΔΨm were compared in APPΔCT15 and WT MEFs. Figure 3(E) shows that ATP level was lower and ΔΨm was higher (Fig. 3F) in APPΔCT15 MEFs compared to WT MEFs.

Previous studies suggested that the transcriptional effect of AICD is mediated via interaction with Fe65 (McLoughlin & Miller, 2008). To further support the notion that AICD regulates PGC-1α expression and to test whether such an effect involves Fe65, we employed an SH-SY5Y neuroblastoma cell line, in which the expression of Fe65 was knocked down (50% downregulation, Fig. 3G). Figure 3(G) shows a significantly lower (21%) amount of PGC-1α mRNA in SH-SY5Y-Fe65KD cells than in SH-SY5YCon cells. Taken together, these results suggest that AICD, via its interaction with Fe65, can regulate PGC-1α expression.

**Figure 4:**
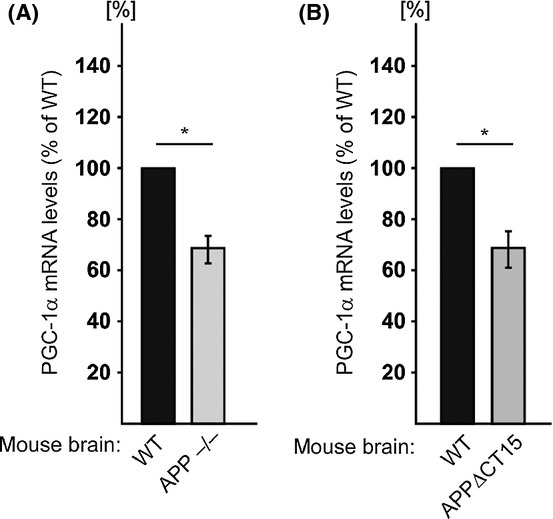
PGC-1α mRNA levels in *APP*^−/−^ and APP∆CT15 mouse brains. Total RNA was prepared from brains of WT, *APP*^−/−^, or APPΔCT15 male mice [*APP*^−/−^ mice (*n* = 4), three mice were 6 months old and one 5 months old. Their corresponding control WT mice (*n* = 4) were 6 months old; APP∆CT15 mice (*n* = 3) were 15, 16, and 17 months old. Their corresponding control WT mice (*n* = 3) were 18 months old]. PGC-1α mRNA levels were determined by real-time reverse transcription quantitative polymerase chain reaction. Values shown are means ± SEM (bars); *P < 0.05 (Student’s *t*-test).

To test whether APP/AICD can also regulate PGC-1α expression *in vivo*, in the brain, we determined and compared PGC-1α mRNA levels in RNA samples prepared from brains of WT, *APP*^−/−^, and APPΔCT15 mice. Figure 4 shows that PGC-1α mRNA levels are indeed lower in the *APP*^−/−^ (31% reduction, Fig. 4A) and APPΔCT15 (31% reduction, Fig 4B) mouse brains compared to WT mouse brains. These results thus suggest that regulation of PGC-1α expression by APP/AICD also occurs *in vivo* in the brain.

## Discussion

In the present study, we tested the effect of PS1 on mitochondria. The following main findings were obtained: (i) deficiency in PSs or AICD impairs the bioenergetic state of MEFs; (ii) PS1 upregulates protein and mRNA levels of PGC-1α; (iii) PS1 upregulates PGC-1α target genes; (iv) PS1-FAD mutations abrogate PS1’s ability to regulate PGC-1α expression; (v) inhibition of γ-secretase activity decreases PGC-1α mRNA levels, and this effect can be bypassed by exogenously added AICD; (vi) APP and AICD, but not Aβ, regulate PGC-1α mRNA levels; (vii) AICD upregulates PGC-1α promoter activity; (viii) the AICD partner Fe65 regulates PGC-1α mRNA levels; (ix) regulation of PGC-1α expression by APP/AICD also occurs *in vivo* in mouse brains. Collectively our results suggest that PS1 regulates PGC-1α expression via APP/AICD and that impairment in this effect may lead to mitochondrial dysfunction.

That PS1 regulates PGC-1α expression was first implied by the PGC-1α regulation of many of the PS1-regulated proteins identified by the SILAC/MS/MS screen [e.g., ATP synthase subunits (Wu *et al*., 1999)]. This notion is supported by the finding that the mRNA levels of PGC-1α and its target genes (e.g., NRF2) were higher in PS1-expressing MEFs than in PS1/2^−/−^ MEFs. These results also indicate that PS1 can regulate mitochondrial functionality as PGC-1α and NRF2 act together to promote nuclear-encoded mitochondrial gene expression (Liang & Ward, 2006). Notably, PS1 may regulate the expression of mitochondrial proteins also independently of PGC-1α, as among the proteins that were upregulated in PS1r MEFs are the solute carrier proteins, whose expression is not known to be regulated by PGC-1α.

The impairment in PGC-1α regulation by the PS1-FAD mutants suggests that modulation of PGC-1α expression may be involved in the pathology of PS1-FAD and perhaps also in sporadic AD. This assumption is in line with previous studies which showed that PGC-1α mRNA levels are decreased in the hippocampus of AD brain (Qin *et al*., 2009; Sheng *et al*., 2012). Furthermore, impairments in PGC-1α were suggested to play a role in other neurodegenerative diseases such as Huntington’s and Parkinson’s diseases (McGill & Beal, 2006; Shin *et al*., 2011).

Our results also show that APP/AICD regulates PGC-1α mRNA levels and that inhibition of γ-secretase activity or PS1-FAD mutations impair PGC-1α expression. These findings, together with a previous study showing that PS1-FAD mutations (including those employed in the present study) alter γ-secretase activity (Kim & Kim, 2008), suggest that the effect of PS1 on PGC-1α expression is mediated via the γ-secretase cleavage product, AICD. This is further supported by the findings that addition of AICD to APPΔCT15 and PS1/2-/- MEFs as well as to SH-SY5Y cells treated with γ-secretase inhibitor enhances PGC-1α mRNA levels and that Fe65 knockdown inhibits PGC-1α expression. Aβ does not seem to regulate PGC-1α mRNA levels, as addition of Aβ peptides to *APP/APLP2*^−/−^ MEFs did not affect PGC-1α expression. We cannot exclude, however, the possibility that other pathways, γ-secretase-dependent pathway or γ-secretase-independent pathway, may also play a role in the effect of PS1 on PGC-1α expression. Our results clearly indicate a role of AICD in PGC-1α regulation. However, APP is only one of many γ-secretase substrates, and therefore, we cannot rule out that other γ-secretase generated, AICD-like peptides are involved. In addition, it is also possible that other PSs-independent regulatory events on PGC-1α transcription and/or protein synthesis/stability may be involved. Further studies are needed to address this issue.

Notably, AICD can affect mitochondrial bioenergetics also by other mechanisms, for example a transcriptional effect of AICD on transgelin (Ward *et al*., 2010) or an effect of AICD on regulation of Ca^2+^ homeostasis, in which a reduction in AICD levels reduces ATP levels and causes hyperpolarization (Hamid *et al*., 2007). Our results showed that PS1 or AICD deficiency reduces ATP levels and enhances mitochondrial membrane potential. The observation that PS1 and AICD deficiency lead to similar mitochondrial dysfunction further supports the notion that PS1 regulates mitochondrial energetic status via AICD. Another mechanism proposed to mediate the effects of PSs deficiencies or PS1-FAD mutations on mitochondrial dysfunction is impairment in mitochondria-associated membrane (MAM) function (Schon & Area-Gomez, 2010). However, whether AICD plays a role in the MAM is still unknown. Together with our current results, it appears that PS1 and AICD affect mitochondria by multiple mechanisms.

**Figure 5:**
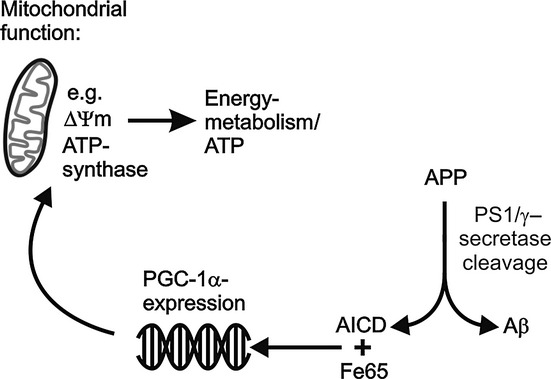
Proposed model for the effect of the PS1/APP/amyloid precursor protein intracellular domain pathway on PGC-1α and energy metabolism.

In summary (Fig. 5), our results suggest that PS1, via its γ-secretase cleavage of APP and generation of AICD, regulates the expression of PGC-1α, a master mitochondrial transcription coactivator. Thus, the PS1/APP/AICD pathway may play a role in regulating the bioenergetics state of cells, and therefore, impairment in this pathway, for example by PS1-FAD mutation, may lead to mitochondrial dysfunction. This, together with other factors, might play a role in AD pathology.

## Experimental procedures

### Reagents

Lipofectamine was purchased from Gibco BRL (Life Technologies, Renfrewshire, UK). Fetal calf serum (FCS) was purchased from PAN Biotech (Aidenbach, Germany) or Thermo Scientific HyClone (Logan, UT, USA). Unless otherwise stated, all other reagents were purchased from Sigma (St. Louis, MO, USA).

### Buffers

The composition of the buffers used in the study is detailed in Supporting Information.

### Cell cultures and mice

Cell lines: DKO MEFs (Herreman *et al*., 2000) reconstituted with empty expression vector pcDNA3.1 (PS1/2^−/−^_,_ PS1/2^−/−^a and PS1/2^−/−^b clones) or with pcDNA3.1 vectors directing the expression of WT human PS1 (PS1r, PS1r.1, PS1r.2, and PS1r.3 clones) or one of the following PS1-FAD mutations: P267S, A285V, T354I, or L392V (Grimm *et al*., 2005); MEFs derived from mice in which the APP locus was replaced by APP mutant which lacks the last 15 C-terminal amino acids (APP∆CT15) (Ring *et al*., 2007); APP- and APLP2-deficient MEFs (*APP/APLP2*^−/−^) (Heber *et al*., 2000) and their corresponding WT MEFs. The latter were derived from the corresponding WT mouse embryos (Ring *et al*., 2007); human neuroblastoma cell line SH-SY5Y control (SH-SY5YCon) and SH-SY5Y cells in which Fe65 was knocked down (SH-SY5Y-Fe65KD) were generated by transfecting SH-SY5Y cells with SureSilencing™ Fe65-shRNA plasmid or with control vector according to the manufacturer’s protocol (SABioscience, Frederick, MD, USA), followed by selection for stable clones with hygromycin B (400 μg mL^−1^). *APP*^−/−^ mice and APPΔCT15 mice were generated as previously described (Ring *et al*., 2007).

### Antibodies

Mouse anti-ATP synthase α monoclonal antibody (Ab) (mAb) (sc-58613) and anti-β-catenin mAb (sc-7963) were purchased from Santa Cruz Biotechnology, Inc. (Santa Cruz, CA, USA). Superoxide dismutase 2 antibody (Ab-13533) was purchased from Abcam (Cambridge, UK). Anti-PGC-1α mAb (4C1.3) was purchased from Calbiochem (EMD Millipore, Darmstadt, Germany). Rabbit anti-APP C-terminal polyclonal antibodies (A8717) were purchased from Sigma-Aldrich (St. Louis, MO, USA). Aβ was detected with W02 mAb (Ida *et al*., 1996).

### Application of Aβ and AICD peptides to the cells

Cells were incubated with culture medium supplemented with a mixture of synthetic Aβ_1–40_ (10 ng mL^−1^) and Aβ_1–42_ (1 ng mL^−1^) (synthesized by B. Penke, Szeged, Hungary) or AICD (KMQQNGYENPTYKFFEQMQN) (2 μm) (GenScript Corporation, Piscataway, NJ, USA) peptides for three passages (9 days).

### Stable isotope labeling with amino acids in cell culture/MS/MS

PS1r and PS1/2^−/−^ MEFs were grown in medium supplemented with isotope-labeled heavy and light amino acids, respectively, followed by preparation of mitochondrial-enriched subcellular fraction. Protein extract of this fraction was then separated by 10% SDS-PAGE followed by In-gel proteolysis and MS analysis.

For detailed description of the SILAC/MS/MS procedures, see Supporting Information.

### Real-time reverse transcription quantitative PCR analysis

Total RNA was isolated using TRIzol Reagent (Invitrogen Life Technologies, Paisley, UK) or the 5 Prime RNA Isolation Kit (DNA Technologies Inc, Gaithersburg, MD, USA) according to the manufacturer’s instructions. Total RNA (1–2 μg) was reverse-transcribed using SuperScript™ First-Strand Synthesis System (Invitrogen) or High-Capacity cDNA Reverse Transcription Kit (Applied Biosystems, Darmstadt, Germany) according to the manufacturer’s protocol. The obtained cDNA was used as a template for RT-qPCR using an ABI 7300 or 7500 Fast Real-Time PCR System (Applied Biosystems, Foster City, CA, USA) and ABsolute™ Blue SYBR Green (ABgene, Epsom, UK) or Fast SYBR Green Master Mix (Applied Biosystems). Relative levels of the different transcripts were quantified using the 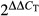 method (Livak & Schmittgen, 2001; Lukasiak *et al*., 2008), and samples were normalized to β-2-microglobulin (B2M) or β-actin. Primers were purchased from Eurofins MWG Operon (Ebersberg, Germany) or Hy Laboratories Ltd. (Rehovot, Israel).

### Treatment with γ-secretase inhibitor

SH-SY5Y cells were incubated for 24 h with the γ-secretase inhibitor L-658.458 (2 μm) (Calbiochem) in DMEM 10% FCS. Afterward, the cells were washed with DMEM and incubated for additional 12 h with 2.5 μm AICD peptide in the presence of 2 μm L-658.458.

### Measurement of intracellular ATP levels

ATP levels in PS1r and PS1/2^−/−^ MEFs were measured using the ATP bioluminescent somatic cell assay kit (Sigma-Aldrich). Luminescence was measured using a Tecan Safire Microplate Luminometer (Tecan, Crailsheim, Germany). The light signal was integrated for 10 s after a delay of 1 s.

### Oxygen consumption

Oxygen consumption was measured using a Clark-type oxygen electrode connected to an Oxytherm unit (Hansatech, Norfolk, UK). Measurements were performed at 37 °C with continuous stirring in 1 mL growth medium. Before each measurement, the medium in the chamber was equilibrated with air for 30 min, and freshly trypsinized cells (2 × 10^6^) were transferred to the respirometer glass chambers. Absolute values based on an oxygen content of 230 nMol O_2_ per 1 mL medium at 37 °C were calculated. After measurement, the cells were collected and protein concentration in each sample was determined. To calculate oxygen consumption rates, mean values of oxygen consumption (nmol min^−1^ mg^−1^ protein) for a 10-min period were obtained. Blank mean value of oxygen consumption rate of medium without cells was measured in every experiment and subtracted from the obtained values of mean oxygen consumption rate.

### Measurement of mitochondrial membrane potential

ΔΨm was measured by TMRM probe (Anaspec Inc., San Jose, CA, USA) using flow cytometry (FACSort; BD Biosciences, Heidelberg, Germany) with an argon laser at 488 nm. *APP/APLP2*^−/−^ and WT MEFs or PS1r and PS1/2^−/−^ MEFs (5 × 10^6^ cells) were incubated with TMRM (10 nm) in the dark at 37 °C for 30 min. The cells were then washed twice with phosphate-buffered saline (PBS), harvested, resuspended in PBS, and subjected to flow cytometry. The mean of the TMRM signal obtained by the flow cytometry analysis was calculated with FACSdiva 6.1 software (BD Biosciences). Background fluorescence was determined in cells treated with TMRM together with the uncoupler carbonyl cyanide m-chlorophenylhydrazone (20 μm).

### Immunoblot analysis

Protein extracts were prepared by radioimmunoprecipitation assay buffer or by lysis buffer A. Total protein extracts were adjusted to the same protein amount per lane (100 μg for β-catenin, superoxide dismutase 2, and ATP synthase α or 20–40 μg protein for PGC-1α) and were separated by 12.5% or 10% SDS-PAGE, respectively, and electroblotted onto supported nitrocellulose. Each blot was blocked for 1 h in Tris-Buffered Saline containing Tween 20 (TBST) buffer containing 5% fat-free milk and then incubated with a primary antibody overnight at 4 °C. Membranes were then washed three times (10 min each) with TBST followed by incubation for 1 h at room temperature with the appropriate second Ab. The blot signals were developed using chemiluminescence reagents. Immunoblots obtained from at least three independent experiments were scanned, and the intensities of the different proteins were assessed by densitometric analysis using ImageJ software (Wayne Rasband, National Institutes of Health, Bethesda, MD, USA).

### Measurements of Aβ uptake

After incubation with the Aβ peptides, the cells were washed three times with ice-cold PBS, then centrifuged (1500 *g* for 5 min), and the resultant cell pellets were lysed at 4 °C in 500 μL lysis buffer B. Aβ analysis in the extracts was performed as described (Ida *et al*., 1996).

### Measurement of AICD uptake by immunofluorescence

MEF∆CT15 cells were seeded on coverslips and incubated for 30 min with AICD peptide (2.5 μm) and the protein delivery reagent Saint-PhD (Synvolux Therapeutics, Groningen, the Netherlands) according to the manufacturer’s protocol. The cells were then washed three times with PBS, fixed with 4% paraformaldehyde in PBS, and stained with anti-APP C-terminal antibody A8717 according to the manufacturer’s protocol. Fluorescent images were captured using a fluorescence microscope (Observer Z1; Carl Zeiss, Jena, Germany) connected to a CCD camera (AxioCam MRm; Carl Zeiss) using Axio Vision (version 4.7.2.0; Carl Zeiss) software and exported to Photoshop (Adobe). Nuclei were detected by staining with the DNA-binding dye 4′,6-diamidino-2-phenylindole (DAPI) (1 μg mL^−1^).

### Assessment of PGC-1α promoter activity

APP∆CT15 MEFs were transfected with PGC-1α 2 kb promoter-luciferase (firefly) reporter (PGC-1α-Luc) (Plasmid 8887; Addgene, Cambridge, MA, USA), promoter-less reporter (pGL3-Basic), or pGL3-Control (Promega, Madison, WI, USA) plasmid together with pRL-CMV plasmid (Promega). Cells were transfected using Lipofectamine 2000 transfection reagent (Invitrogen) according to the manufacturer’s instructions. pRL-CMV (Renilla luciferase) was used for assessing transfection efficiency. Twenty-four hour after transfection, the cells were incubated for 90 min with AICD peptide (2.5 μm) and the protein delivery reagent Saint-PhD (Synvolux Therapeutics) according to the manufacturer’s protocol. Luciferase assay was then performed with the Dual-Glo® Luciferase Assay System (Promega) according to manufacturer’s instructions. Promoter activity was normalized for transfection efficiency by dividing firefly luciferase activity by Renilla luciferase activity, followed by subtracting the basal activity of the promoter-less reporter, pGL3-Basic.

### Statistical analysis

Data are expressed as mean values ± SEM and are analyzed using Student’s *t*-test. Differences were considered statistically significant at *P* < 0.05.

## Supporting Information

**Fig. S1** Aβ peptide is taken up by *APP*^−/−^*APLP2*^−/−^ MEFs.

**Fig. S2**AICD peptide is taken up by APPΔCT15 MEFs.

**Fig. S3** The effect of AICD incubation on PGC-1α mRNA levels in SH-SY5Y cells.

**Table S1.** ATP synthase subunits detected by the SILAC screen.

**Data S1** Experimental procedures and legend of Fig. S1.
