# Supplementary figures and images for: Upregulation of PGC-1α expression by Alzheimer’s disease-associated pathway: presenilin 1/amyloid precursor protein (APP)/intracellular domain of APP
Source: Aging Cell. 2013 Dec 17;13(2):263–72. doi: 10.1111/acel.12183 (PMC4331788; doi:10.1111/acel.12183)

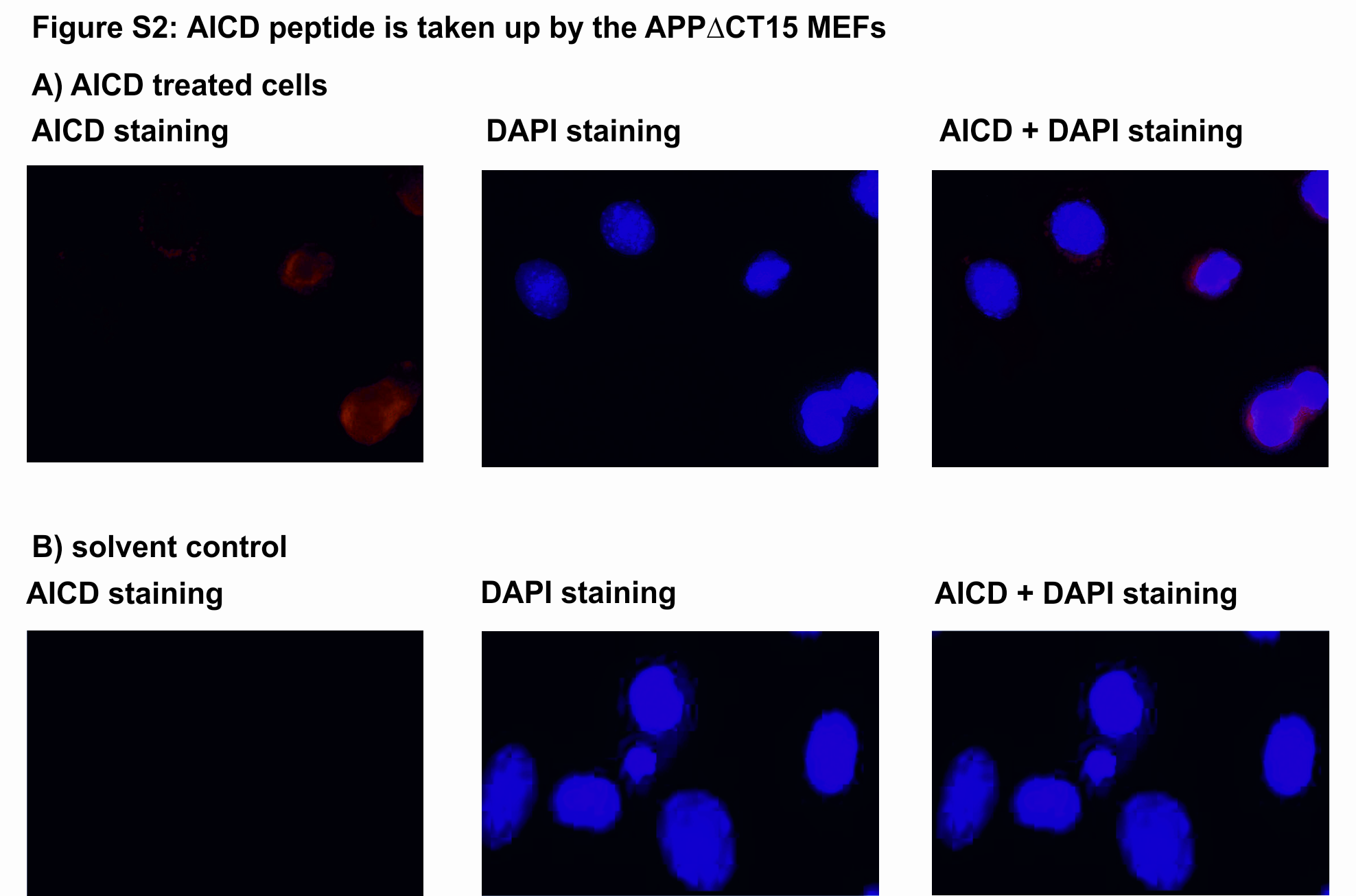

Supplement: Supplementary file 2 — Fig. S2AICD peptide is taken up by APPΔCT15 MEFs. [file acel0013-0263-sd2.tif]
