# Supplementary material for: Upregulation of PGC-1α expression by Alzheimer’s disease-associated pathway: presenilin 1/amyloid precursor protein (APP)/intracellular domain of APP
Source: Aging Cell. 2013 Dec 17;13(2):263–72. doi: 10.1111/acel.12183 (PMC4331788; doi:10.1111/acel.12183)
Supplement: Supplementary file 4 — Table S1. ATP synthase subunits detected by the SILAC screen. [file acel0013-0263-sd4.docx]

**Table S1.** ATP synthase subunits detected by the SILAC screen

| **DKOE/PS1 %** | **ATP**  **synthase subunit** |
| --- | --- |
| 81  79  78  75  62  61  46 | ATP synthase subunit O  ATP synthase subunit β  ATP synthase subunit b isoform1  ATP synthase subunit α  ATP synthase subunit γ mitochondrial precursor  ATP synthase subunit γ  ATP synthase subunit δ |

The results are expressed as percentage of light (DKO) from heavy (PS1r) peptides of each protein.
