# Supplementary material for: Upregulation of PGC-1α expression by Alzheimer’s disease-associated pathway: presenilin 1/amyloid precursor protein (APP)/intracellular domain of APP
Source: Aging Cell. 2013 Dec 17;13(2):263–72. doi: 10.1111/acel.12183 (PMC4331788; doi:10.1111/acel.12183)
Supplement: Supplementary file 5 — Data S1 Experimental procedures and legend of Fig. S1. [file acel0013-0263-sd5.doc]

**Supporting Information**

**Experimental Procedures**

**Materials**

Second goat anti-mouse or goat anti-rabbit IgG peroxidase conjugate was purchased from Jackson ImmunoResearch Laboratories (West Grove, PA, USA).

The Fe65-shRNA plasmid (SABioscience, Frederick, MD, USA) contain the Fe65 sequence, 5'-AAGGCTTTGAGGATGGAGAAT-3' and the control vector contained the sequence randomized, 5'-GGAATCTCATTCGATGCATAC-3'.

**Cell lines**

All cell lines were cultured in Dulbecco's modified Eagle's medium (DMEM) supplemented with 10% FCS at 37°C and 5% CO_2_. The culture media of DKOE MEFs, PS1r MEFs and MEFs with PS1-FAD mutations were supplemented with Zeocin^TM^ (300 µg/ml) (Invitrogen, Karlsruhe, Germany), and those of SH-SY5Y-Fe65KD cells were supplemented with hygromycin B (400 μg/ml) (PAN Biotech, Aidenbach, Germany).

**Buffers**

Mitochondrial homogenization buffer contain: 200 mM mannitol, 70 mM sucrose, 1 mM EGTA, 10 mM HEPES pH 7.5, 5 mM MgCl_2_, and 1 mM DTT, as well as the protease inhibitors aprotinin (0.15 U/ml), leupeptin (5 μg/ml) and phenylmethylsulfonylfluoride (1 mM). Radioimmunoprecipitation assay buffer contain: 1% NP-40, 1% sodium deoxycholate, 0.1% SDS, 150 mM NaCl and 50 mM Tris base, pH 8.0. Lysis buffer A contain: 20 mM sodium phosphare (pH 7.5), 5 mM EDTA and 5 mM β-mercaptoethanol).

Lysis buffer B contain: 150 mM NaCl, 50 mM Tris-HCl pH 7.5, 2 mM EDTA, 1% Nonidet P40 and 1% Triton X-100).

TBST buffer contain: 10 mM Tris base, 150 mM NaCl, and 0.05% Tween 20.

**Primers**

The following primer sequences were used for RT-qPCR analysis: Murine: **Slc25a13**: 5´-TTCTATTGCTGGAGCTGTCG-3´ and 5´-TATACATGAGCTCGCCCACA-3´; **Slc25a1**: 5´-CTCGACAGCAGGAGAGGACT-3´ and 5´-GGATGAACTTCACCTTGATGG-3´; **Slc25a11**: 5´-CTTCTGAAAGCCCTGATTGG-3´ and 5´-GAAGCCGACCATCAGCAG-3´; **Slc25a5**: 5´-CAGCTGGATGATTGCACAGT-3´ and 5´-ATCAGTTCCTTTGCGTCCAG-3´; **Slc25a12**: 5´-CTTCAGAGGCAGCAATCTCC-3´ and 5´-CCCACAGCTCCAGCAACT-3´; ATP synthase subunit gamma (**Atp5c1)**: 5´-GAAGCCTGCCCGAGTGTA-3´ and 5´-CACACCAATAATGAGGTGCTTC-3´; **Atp5h**: 5´-CAATTGGAAATGCCCTGAAG-3´ and 5´-CATTGGCCCTGTAGTAAGCC-3´; ATP synthase subunit α (**AtpA)**: 5'-TCTCCATGCCTCTAACACTCG-3' and 5'-CCAGGTCAACAGACGTGTCAG-3'; **Pcx**: 5´-CACAGTGGACACCCAGTTCA-3´ and 5´-CCATGACATGTCCGAGGTAA-3´; **Iars2**: 5´-TCTCCCTCATCAAGGACTGC-3´ and 5´-ACTCCAACTTCGGAGGAGGT-3´; **Aldh2**: 5´-TCAATATCGTTCCCGGATTC-3´ and 5´-GATTAGGTGACCAACCTCCGT-3´; **Cyc1**: 5´-AGGCTGCAAGAGCTGCTAAC-3´ and 5´-AGTAGCCAGTGAGCAGGGAA-3´; **Sod2**: 5´-GGAGAACCCAAAGGAGAGTTG-3´ and 5´-GAACCTTGGACTCCCACAGA-3´; **Clic4**: 5´-GTGCCCACCCAAGTACCTAA-3´ and 5´-CGCTTCATTAGCCTCTGGTC-3´; **PGC-1α**: 5´-GACCCCAGAGTCACCAAATGA-3´ and 5´-TTCCAGAGAGTTCCACACTTA-3´; **NRF2:** 5'-TCTTGGAGTAAGTCGAGAAGTGT-3' and 5'-GTTGAAACTGAGCGAAAAAGGC-3'; **B2M**: 5'-CATGGCTCGCTCGGTGACC-3' and 5'-AATGTGAGGCGGGTGGAACTG-3'. **β-Actin**: 5´-CCTAGGCACCAGGGTGTGAT -3´ and 5´-TCTCCATGTCGTCCCAGTTG -3´. The following human primer sequences were used: **PGC-1α**: 5'-GTTCAAGATCGCCCTACAGC-3' and 5'-CCCTCTCAGACTCTCGCTTC-3'; **Fe65** mRNA levels in SH-SY5Y-Fe65KD cells were assessed with the following Fe65 primer pairs. Pair 1: 5'-TCTTGCACCAGCAGACAGAG-3' and 5'-CAGCCATGATGAATGCAAAC-3'; pair 2: 5'-TTTGGAAGGATGAACCCAGT-3' and 5'-AAGCTTCTCCTCCTCTTGGG-3'; pair 3: 5'-GCTCTAAGATCATGGCCGAA-3' and 5'-GGAATTCCACTTGGAAAGGG-3'.

**Stable isotope labeling with amino acids in cell culture (SILAC)**

PS1r MEFs were grown for seven passages in lysine- and arginine-free RPMI 1640 medium (Biological Industries Ltd., Beit Haemek, Israel) with 10% dialyzed FCS (JRH Biosciences, Lenexa, KS, USA) supplemented with isotope-labeled "heavy" L-arginine-^13^C6,^15^N_4_ hydrochloride ([^13^C_6_,^15^N_4_]arginine; 1.15 mM) and L-lysine-^13^C_6_,^15^N_2_ hydrochloride ([^13^C_6_,^15^N_2_]lysine; 0.274 mM), whereas PS1/2^−/−^ cells were grown with normal "light" amino acids.

**Preparation of mitochondrial-enriched subcellular fraction**

10^7^ labeled PS1r or PS1/2^−/−^ cells were trypsinized and then pelleted by centrifugation at 600 *g* for 5 min. Pellets were washed once in phosphate-buffered saline (PBS), resuspended in 1 ml ice-cold PBS and then pooled in an Eppendorf tube and pelleted by centrifugation at 1200 *g*, at 4°C for 5 min. The pellet was resuspended in 1 ml of ice-cold mitochondrial homogenization buffer, incubated for 10 min on ice and then homogenized by about 10 strokes with a Dounce homogenizer size A, followed by 10 strokes in Dounce homogenizer size B. The homogenate was centrifuged at 1500 *g* at 4°C for 5 min and then the supernatant was centrifuged at 10,000 *g* at 4°C for 10 min to pellet crude mitochondria. The pellet was resuspended in 40 µl ice-cold mitochondrial homogenization buffer and then proteins were extracted by sonication. Proteins (75 µg) were separated by 10% SDS-PAGE.

**In-gel proteolysis and mass spectrometry (MS) analysis**

Following separation of the labeled and unlabeled proteins, the gel was cut into five slices and the proteins in each slice were proteolyzed "in gel". The resulting peptides were resolved by on-line reverse-phase nanoscale capillary liquid chromatography (LC) and analyzed by electrospray MS/MS as described previously . The MS data were analyzed using Sequest 3.31 software (including Extract_msn for peak list generation) (J. Eng and J. Yates, University of Washington and Thermo Finnigan, San Jose, CA, USA) by searching against the mouse section of the NCBI NR database (updated to Jan 2008). Quantification was performed as described previously using PepQuant (Thermo Fisher Scientific) to measure the peak area of each identified peptide with both heavy (H) and light (L) isotopes and a mass tolerance of 0.01 Da using combined scans. The L:H ratio of a protein was determined as an average of the ratio of its peptides.

**Figures S1-S3**

**Figure S1.** Aβ peptide is taken up by the APP^-/-^/APLP2^-/-^ MEFs.

APP^-/-^/APLP2^-/-^ MEFs were incubated with ddH_2_O (−) or Aβ peptides (+) for 9 d. After incubation, the cells were washed three times with ice-cold PBS, then centrifuged (1500 *g* for 5 min) and the resultant cell pellets were lysed at 4°C in 500 µl lysis buffer (150 mM NaCl, 50 mM Tris-HCl pH 7.5, 2 mM EDTA, 1% Nonidet P40, 1% Triton X-100). Aβ analysis in the extracts was performed as described previously .

**Figure S2**: AICD peptide is taken up by the APPΔCT15 MEFs.

**
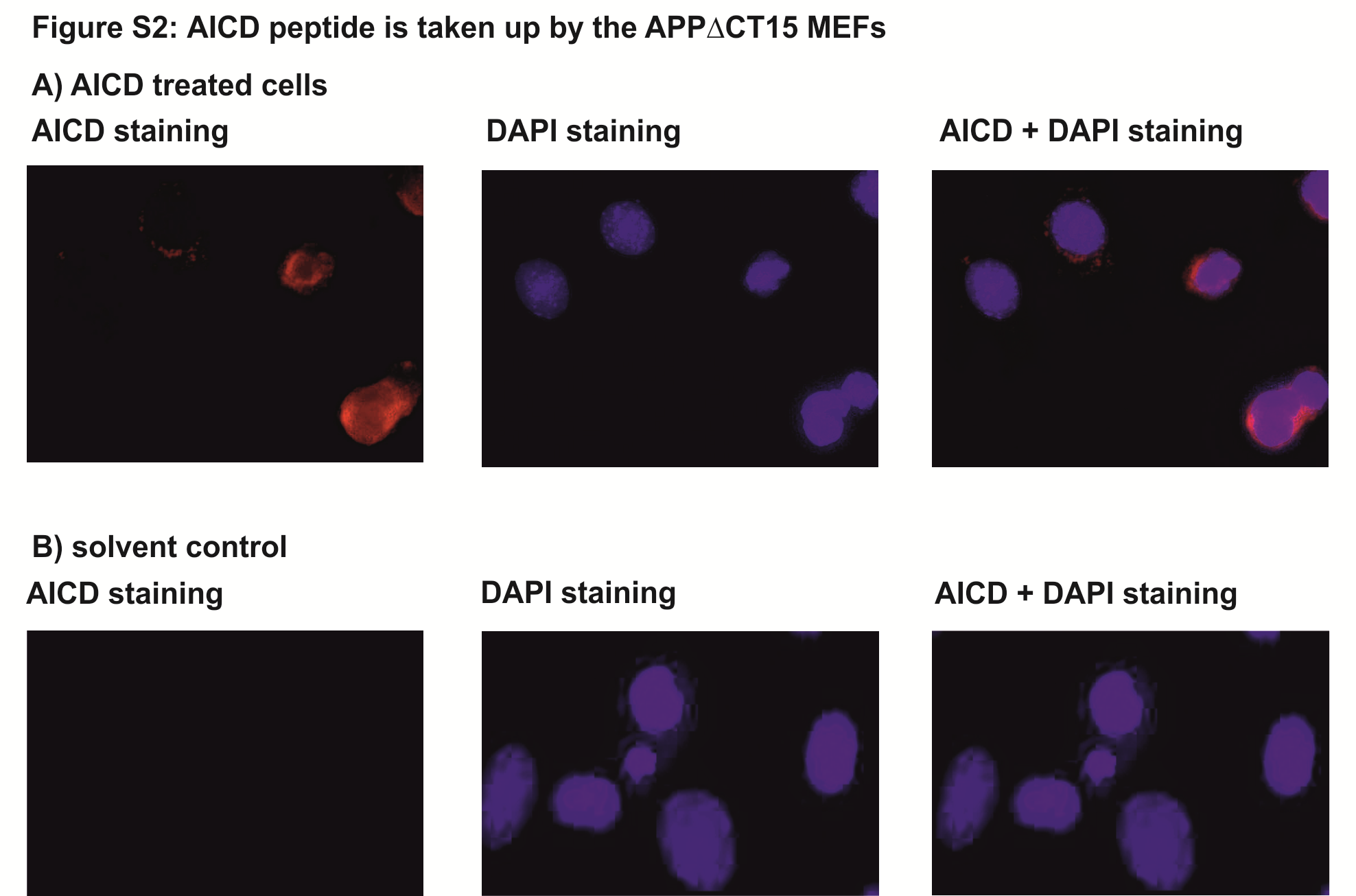
**

APPΔCT15 MEFs were incubated with the AICD peptide (2.5 µM) (A) or solvent (B) for 30 min and then intracellular AICD was detected by immunofluorescence staining as described in the Experimental Procedures.


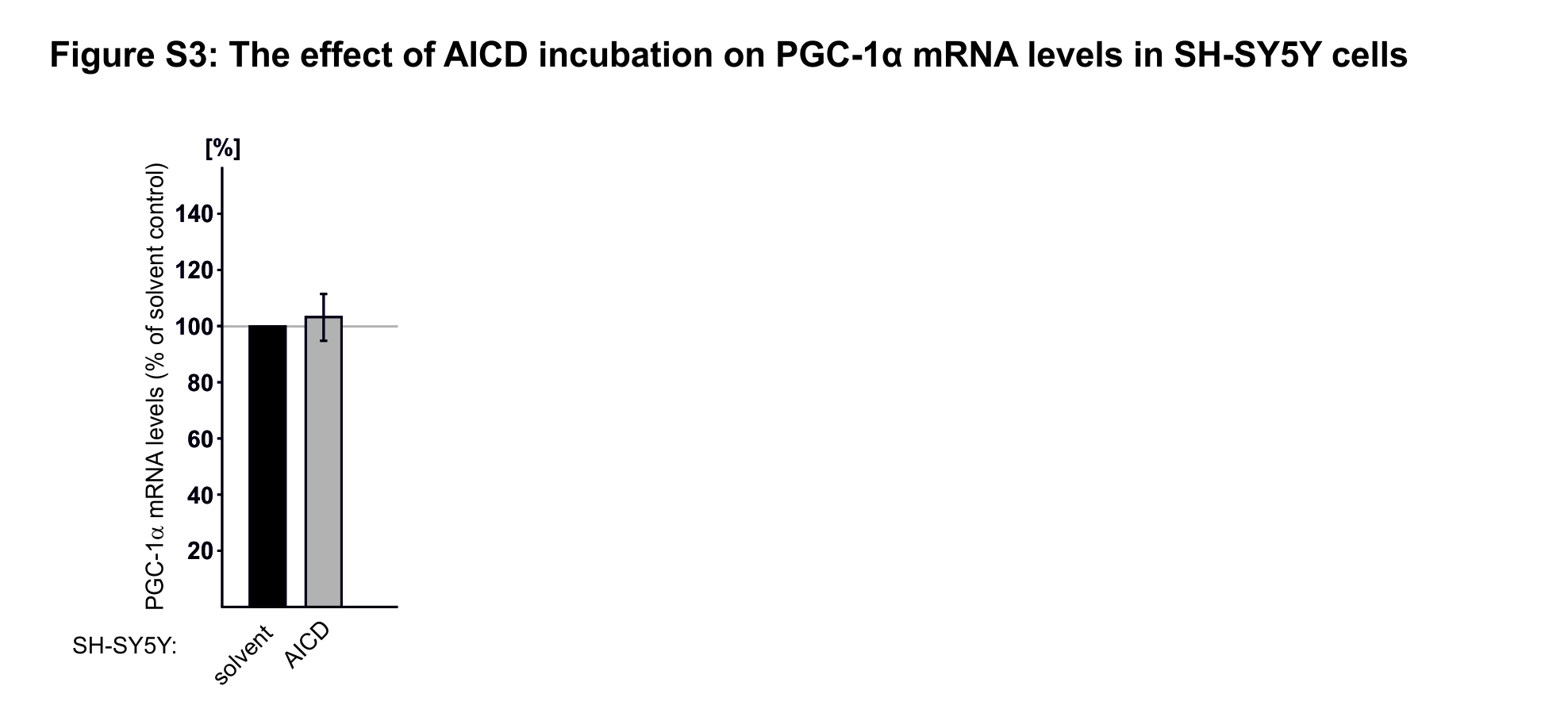
**Figure S3**: The effect of AICD incubation on PGC-1α mRNA levels in SH-SY5Y cells. SH-SY5Y cells were incubated with Saint PhD (solvent) or with 2.5 µM and Saint PhD and AICD for 12 h after which PGC-1α mRNA levels were determined by RT-qPCR as described in the Experimental Procedures. No significant difference was observed between the two treatments (*n* = 3, student´s *t*-test)

**References**

Hor S, Ziv T, Admon A, Lehner PJ (2009). Stable isotope labeling by amino acids in cell culture and differential plasma membrane proteome quantitation identify new substrates for the MARCH9 transmembrane E3 ligase. Mol Cell Proteomics. 8, 1959-1971.

Ida N, Hartmann T, Pantel J, Schroder J, Zerfass R, Forstl H, Sandbrink R, Masters CL, Beyreuther K (1996). Analysis of heterogeneous A4 peptides in human cerebrospinal fluid and blood by a newly developed sensitive Western blot assay. J. Biol. Chem. 271, 22908-22914.
